# Geology of Ohio

**Published:** 1833

**Authors:** John L. Riddell

**Affiliations:** Worthington, Ohio


### Art. VI.—Geology of Ohio.

*Observations on the Geology of the central parts of the State of Ohio, by* John L. Riddell, A. M.

I must premise, that I am not aware any one has given to the scientific world, very extended observations on the geology of this part of Ohio: and with candor I must confess, that my own opportunities for the collection of facts on this subject, have been quite limited.

The expanse of country constituting the central portions of this State, seems to form a continuous and level plain, which is frequently intersected by valleys and ravines, that correspond with the water courses; but no where are these depressions or gorges to be found of any remarkable depth. In no country, perhaps, can the formation of valleys be more satisfactorily ascribed to the slow, but certain agency of running waters. Many thousand years, perhaps, have witnessed their gradual excavation, by the transporting power of floods.

Without descending to nice distinctions, we may notice three well-marked varieties of soil, which correspond with what some geologists have denominated, the *Modern Alluvion*, the *Ancient Alluvion*, and the *Analluvion*.*

* Eaton.

The *Modern Alluvion* comprises those rich bottom lands, which border the low banks of the rivers and creeks. The *Ancient Alluvion* is altogether the most predominant. The materials of which it is composed, seem to have been tranported by rapid and powerful currents of water; and the territories over which it is spread, are such as to prohibit the idea of referring its origin to causes analogous to those now in operation. The great number of primitive bowlders which occur in this formation, constitute its most remarkable feature. They may be observed of various sizes, from the smallest pebble, to the dimensions of huge, irregular, partially rounded masses, of many tons weight. They are particularly abundant at the bottom of valleys, where the earth has been gradually washed away. Among them can be observed almost every variety of granite, gneiss, hornblende, sienite, quartz, &c. Very seldom indeed, can specimens of the more soft or fissile minerals be found; as mica slate, steatite, or chlorite; and their absence accords well with the supposed alluvial origin of this formation: for the mechanical violence which these bowlders and rocky masses must have suffered, in being transported to their present situation, would have been amply adequate to reduce the softer portions to a fine state of comminution. This variety of soil then, with its mineral contents, evidently belongs to the "*erratic block group*" of De la Beche. It is not a peculiarity of this particular region, or this State; but traces of it can probably be observed in nearly all parts of our country, as well as in other portions of the globe. As suggested by De la Beche and others, it was anciently produced, in all probability, by a powerful and overwhelming inundation of waters, rushing along from the regions of the north.

The *Analluvial detritus* (analluvion of Eaton) occurs here and there; especially where the pyritous shale, a rock that easily disintegrates, happens to be uppermost. In such localities, the ordinary action of meteoric agents on the slate, cooperating with the spontaneous changes which the plentifully disseminated pyrites undergoes, results in reducing the whole mass to fine incoherent particles, having much the appearance of ordinary earth. This kind of soil is by no means abundant.

The indurated rock strata consist of *Limestone*, *Graywacke* or sandstone, and *Pyritous shale:* and in descending, they overlay each other in the order I have enumerated them.

The *Limestone* is generally of a bluish gray colour, and not very remarkable for its compactness. In its upper portions there is a plentiful occurrence of hornstone, either disseminated in nodules, or forming distinct and limited strata. In many places the waters have worn deep channels in the limestone rock; and the precipices thus formed, are oftentimes bold and impressive in their appearance. And what sometimes contributes to give additional interest to these gorges, is the occasional appearance of dark, rude caverns, overlooking the waters below; as can be seen in the vicinity of Dublin on the Scioto. The mass of this rock seems, in a great measure, made up from the *exuviæ* of marine animals. It is almost impossible to find a solitary hand-specimen of the limestone, in which the impress of numerous crustaceous and radiated animals cannot be distinctly traced. *Encrinites*, *Productæ*, and *Orthoceratites*, are perhaps most abundant. The virtuoso, who is fond of collecting organic remains, will find very good specimens of *Encrinus*, *equicyclus*, *E. transversus*, *Orthocera circularis*, *O. striata*, *Cyathophyllum vesiculosum* and *Columnaria sulcata* (*Eat.*) He will meet with an abundance of *Bellerophon cornu-arietis*, (*Sowr.*) and several other fossils resembling it: now and then a perfect *Echinus* may be found, still more rarely the *Calymene Blumenbachii?* (*Al. Brogn.*)

So far as my observations have extended, the graywacke and clayslate do not occur in a uniform order of superposition; but where they are seen in connexion, it generally happens that the uppermost strata are sandstone. In some localities, thin strata of this rock can be observed to make a number of alternations with the slate.

The *Graywacke* of this region is characterized by a hue of light gray, inclining to blue or green. Like the equivalent rock of other countries, it consists apparently of quartzose particles united by an aluminous cement. It is generally so soft, of such an even structure, and so free from cracks and hard spots, that it may be easily wrought; and when fashioned into an arch, or a tombstone, it has a very neat and appropriate appearance. In many localities, its consistence is such as to render it well adapted for grindstones. This rock not unfrequently contains thin laminæ of mineral coal, strongly impregnated with bituminous matter: and the occasional occurrence of imperfectly preserved relics of tropical plants, reminds the observer of the prevalent and very reasonable opinion, that the temperature of these northern latitudes, in very ancient times, was much higher than it is now.

The *Pyritous shale* or clay-slate, of some, graywacke-slate, aluminous schist, pyritiferous slate &c. of others, is so universally known, as to render description almost unnecessary. It generally has a dull shade of blue, is chiefly aluminous in its composition, and is almost destitute of grit. It is naturally rather soft, and when freely exposed to the air, is extremely apt to crumble into small fragments. The disintegration is essentially, if not entirely, owing to the large quantity of disseminated iron pyrites. This mineral, which bears a delusive resemblance to gold, is often met with in masses of several pounds weight. It occurs in profuse abundance in smaller pieces, and forms, no doubt, a share of the more intimate composition of the slate. As it is a bi-sulphuret of iron, (consisting of sulphur two equivalents, iron one,) the spontaneous changes which it undergoes when exposed to air, in contact with the alumina of the slate, give rise to the formation of crude alum (sulphate of alumina) and copperas, (proto-sul[illegible]te of iron); and so very common and plentiful are these natural products, that bushels may be easily collected in very many localities. It must be obvious to the manufacturing chemist, that the copperas and alum of commerce, might be prepared here with unusual advantage; the country around yielding all the materials requisite in the preparation.

The most remarkable feature of the pyritous shale, is the frequent occurrence of solid imbedded *globes*, varying from the size of a mere pebble, to that of a sphere ten feet in diameter; and there are localities a few miles from Worthington, where I think they can be found to exceed that measurement. One of the situations to which I refer, is a deep and tortuous ravine, two miles north of this village, and opening into the valley of the Oolentangy river. The forest-clad cliffs of this singular gorge, generally maintain an altitude of seventy or eighty feet, and are made up almost entirely from the pyritous shale. The most of these globes are met with about fifty feet above the level of the Oolentangy; and I can assure those who never saw them, that they here present a spectacle, unique and strange: for one can at first, with difficulty persuade one's self, that they are exclusively the work of nature; so strikingly do they resemble the warlike contrivances of man. Were it not for their gigantic and variable dimensions, one could easily imagine they were cast in furnaces, and shot into the banks from loaded cannon. Indeed they give an air of enchantment to those solitudes, which is not a little heightened in damp winter weather, by the luxuriant growth of rein-deer moss, (*Cenomyce rangiferina*,) that fixes itself on the declivities of the disintegrating slate. Very many of the globes lie loose in the bed of the stream; some of them entire, and some (owing, perhaps, in many instances, to an original want of integrity,) in scattered fragments.

I suspect, from what I can learn, that these strange productions of the earth may be met with in various and distant parts of the State. Dr. Hildreth informs me, that in a visit to the Barrens, west of the Scioto river, he observed the same mineral appearance in the banks and bed of Deer creek, which are entirely composed of loose slate, containing considerable sulphuret of iron. "The same formation" he observes in a letter, "continues, without doubt, from Worthington to Brush creek in Scioto county, near the Ohio river, there rising into considerable hills, and affording alum and copperas in abundance; and immense numbers of these globular bodies, of all sizes, from a nut to a hogshead. As the slate decomposes, they tumble out and roll to the foot of the hill." Col. Kilbourne, of Worthington, states that he has seen these stony spheres, on the banks of Huron river, which empties into Lake Erie. They are likewise plenty in the neighborhood of Newark.

As regards their structure, a few appear equally solid throughout, but most of them are much less compact within. The soft and fragile interior of many, contains a nucleus of equal hardness with the external crust; while not a few are entirely hollow, with the exception of containing a disconnected, hardened core. The traveller, on his way from Columbus to Sandusky, can observe an interesting appearance presented by these spheres in place, two miles and a half north of Worthington, a few rods below where the turnpike bridge crosses a stream of water. The ledge of slate which borders the stream, at this place is fifteen or twenty feet high; and two or three yards from the bottom, five of these concretions may be seen, seemingly just ready to roll out of their shaly beds. They are situated in a line, and occupy, apparently, the same horizontal plane. Four of them (being of equal size) are about two feet and a half in diameter, and near two rods distant from each other. Of these, three are so broken as to present a hollow interior, while one only is entire. The remaining one is smaller than its fellows, and considerably fractured. These bodies, in general, are completely globular, yet many of them have the form of an oblate spheroid; the shorter diameter, when in place, being vertical. They frequently have raised lines passing around them horizontally, at short intervals, like parallels of latitude. These lines, and the intervening spaces, point out their connexion with the laminæ of slate. I possess one, four inches in diameter, encrusted entirely with pyrites, and completely encircled with a raised equatorial ridge, which gives it wholly the appearance of being a work of art. Two spheres, of equal or unequal dimensions, are often seen to coalesce and form a single body, each retaining its globular form, with the exception that the quantity of stony matter is increased about the line of junction. If a recent fracture of the solid exterior portion be examined, a distinct fibrous structure can be observed, running from the surface directly towards the centre. If the fragment be held in a proper position with respect to the light, a glimmering lustre is apparent, owing to the reflection of light from crystalline laminæ, that traverse the radiating fibres at an angle near 45 degrees. Such specimens generally have an appearance similar to dark, fine-grained basalt. Taking the mean of a few trials, I make the specific gravity of this portion, 3.99, snow-water being assumed as unity. I have in my possession, a perfect sphere, less than an inch in diameter, the surface of which is entirely covered with cubical crystals of iron pyrites. It was thrown up in digging a well, from a depth of twenty feet. The interior of these spheres, is more variable in its character and composition: it is generally rather soft, and of a light gray, earthy appearance, frequently containing crystals of calcareous spar. The flexion and displacement of the layers of slate, where these bodies occur, are such, in my opinion, as clearly to indicate the relative antiquity of each. The arrangement is such as would probably have been effected, by the gradual increase of the sphere from a small nucleus; for the layers of slate mostly curve around the globe, both above and below; yet those laminæ which strike the surface at nearly right angles, unite and lose their continuity on the globe. They are frequently invested with a thin coating of iron pyrites, possessed like the proper substance of the globe, of a fibrous structure. Basalt is said sometimes to assume the globular form, but I am not aware of its ever being associated with the clay-slate, in such isolated masses.

Respecting the origin of these bodies, I should perhaps be prudent to remain silent, and leave others to determine. With due deference, however, to our eminent geologists, I will submit the following opinion: It seems to me that these concretions have been formed by the "silent though powerful" agency of electricity. In what manner the galvanic arrangement is made up, I will not presume to decide. It may be by the numberless alternations of pyrites and slate, or by the contact of different rock strata, on a gigantic scale. It is probable that the pyritous shale undergoes a kind of transformation; that the elements composing it are merely aggregated in a more solid and durable manner. But this I confess requires much further investigation. Consistently with such views, I cannot but regard these imperceptible operations, as going on at the present time: and in my opinion, the unfinished, or rudimental condition, in which many of these productions seem to be, gives no small degree of plausibility to the idea. This transition state, from the fissile shale to the indurated sphere, is well exemplified in an excavation for the turnpike, near Kinnear's tavern, midway between Columbus and Worthington.

In conclusion, I hope that some professed geologist will give these subjects a share of his attention.

*Worthington, Ohio, Nov.* 9, 1833.

### Editorial Note.

It will be recollected that the title of our Quarterly embraces the word *physical.* We consider it honorable to the profession, that in all new countries, where the physical sciences are under the necessity of depending on other branches of philosophy, they should have selected Medicine. In truth, the medical and physical sciences belong to one great department of human knowledge—*that of nature.* The physiology of man, is but a branch of general physiology; and to be well known, the latter must be studied. Animals and plants are intimately connected with the earth—drawing from it their subsistance, and modified by it, in many of their characters and properties. Not a few of the diseases which arise in the former, especially in man, are the effect of its waters and its exhalations. Thus geology and mineralogy contribute many aids to the study of ætiology; and the medical history of no district of country, can be regarded as complete, unless it embrace an account of the soil and springs, with a general statement of the rock formations immediately beneath. Still further, there is a propriety in our knowing what the particular region in which we practise the profession, can contribute to the materia medica. Under every aspect, therefore, in which we can view this matter, it seems admissible to render a *medical* journal, in a new country, the vehicle, to a certain extent, of papers on its Mineralogy, Meteorology, Botany and Zoology. Such are the views, which have induced us to publish the interesting paper of Mr. Riddell, on the geology of the central parts of Ohio; and we would take this opportunity, to invite from others, in the valley of the Mississippi, contributions of a similar kind, or on other branches of its physical history.

Mr. Riddell's observations on the rolled pebbles and blocks of primitive rock, which lie scattered over the central parts of Ohio, have reminded us of some of our own inquiries and speculations on the same subject, nearly twenty years ago. They are contained in a letter to the late Abbe Correa, our Minister from the Court of Portugal, and were published in the 2d volume of the new series of the Transactions of the American Philosophical Society, printed in 1825. As a supplement to Mr. Riddell's paper, we shall venture to make the following extract:

1. Fragments of fossil wood, which have been dug up at various depths, from ten to ninety feet. They are not mineralised, and *appear* to have belonged to trees of the same species with some of those which grow in our existing forests. I have not detected among them any tropical plants.

2. Grey, siliceous, and calcareous sand, which composes a great portion of the plain, and has probably resulted from the destruction of arenaceous limestone rocks, many strata of which exist in this country to the north and east.

3. Veins of blue and yellow clay, afforded no doubt by the marl, which in many parts of the limestone tracts, separate the laminæ of that formation.

4. Rolled and angular fragments of blue shell limestone, detached from the strata of the surrounding hills. Many of these are large tabular masses, and seem to have been brought only a short distance. None of them indeed could have come down the river more than one hundred miles, as this variety does not extend beyond that limit to the east. They are very numerous.

5. Polished *debris* of that kind of grey sandy limestone which the Scioto and Little Miami rivers traverse near their sources, and which have evidently been rolled hither by the copious streams that formerly flowed in the valleys of those rivers. These fragments are as numerous as the last.*

6. Rubble and boulders of grey variegated and micaceous sandstone, with minute fragments of coal, aluminous slate, and shivers, generally rendered smooth by attrition. These are from the country up the Ohio, where such strata are found *in place*. They are not so numerous as the two last.

7. Foreign or adventitious *debris*, consisting of granite of different colours, *gneiss*, *micaslate*, *hornblende*, *sienite*, *wacke*, *porphyry*, *trap*, *jasper*, *petrosilex*, *flint*, *agate*, *quartz*, and various other ancient species. These are of every size from small gravel to *boulders* fit for street and court paving. They are not angular, but have suffered attrition, until the distinctive characters of many fragments are almost obliterated, and a fracture must be made before they can be known. They are blended intimately with the other stony wreck, and have not hitherto been found to occupy any distinct bed. The source of this *debris* of primitive and transition strata can be best ascertained by tracing its distribution over the country. I have observed pebbles of this kind on the Hudson river at West Point, where the plain is composed in part of rolled fragments.

But as they are in general larger than those found on the Ohio, less worn and polished, have a greater proportion of mica slate pebbles, which do not seem to bear rolling to a great distance, and are mixed with rubble from the sand stone mountains of Katskill, they should, I think, be considered as being detached from the adjacent primitive rocks. An additional reason for this opinion may be drawn from the silence of our excellent friend Professor Mitchill, as to the *debris* of ancient strata among the alluvion of the upper parts of the valley of this river.† It seems probable then that the currents which rolled and polished these fragments, did not extend laterally as far as the basin of the Hudson. We do not however depart from it westwardly but a short distance, before we meet with the ruins of primitive strata. An observing traveller, Mr. J. C. Warren, informs me that he saw them on the Chenango, a branch of the Susquehanna, and observed them near all the streams in passing thence by the town of Erie on the lake, and along the Allegheny river to Pittsburg. Mr. Thomas Nuttall had previously noted the same thing during a journey in which he visited most of the principal rivers and lakes in the western part of New York. So much for the north-east. To the south-east and south, the valley of the Ohio seems to constitute the boundary of this *debris*. Beyond that river, there are indeed but few water-worn pebbles of *any* kind; and the narrow alluvions of the streams are generally argillaceous. I have travelled over most of the northern and north-eastern parts of Kentucky, and do not now recollect to have seen any foreign rolled pebbles. Mr. Warren has visited the northwestern portions of the same State, and at my request was particularly attentive to this point, but did not discover any. The acute and

*These, on account of the whiteness of the lime into which they burn, are collected and used for that purpose.

† See Medical Repository, Vol. I.

observing Mr. Nuttall travelled through the centre of the same State from north to south on foot, but after leaving the valley of the Ohio opposite Cincinnati, he found none of these fragments until he approached the French Broad, in Tennessee, whose source is in a primordial formation. To the west and south-west, the currents of the great valley have transported the *debris* much farther. Mr. Nuttall has traced it only to the mouth of the river St. Francois; but my late lamented friend Dr. Goforth met with it in the form of gravel, not many miles above Natchez. To the north-west, it was found by Mr. Nuttall on the Missouri, as far as the great bend at the Mandan villages, beyond which he did not ascend. From the mouth of this river to Erie in Pennsylvania, the same persevering naturalist met with it on the shores of all the rivers and lakes which he visited, and from the information which he has kindly afforded me by letter, it appears that this *debris* is larger and in greater quantities in that region than this. Within the limits here sketched, it is found in the vicinity of all the streams, and forms, with the ruins of the surrounding strata, the bases of all the fertile and level prairies. We are hence, I think, justified in the conclusion, that its origin was in the north, and that it was brought and deposited on the surface of this country by currents which in ancient times flowed from beyond the lakes to the Gulf of Mexico, and of which it may be regarded as the sign and effect.

A more recent formation than many of the alluvial beds contained within the limits just defined, is the stratum of loam spread over the surface of our hills and valleys in an *overlying* position. This appears to be the same that in the north of Europe is denominated *geest*,* and which Mr. De Luc considers as the last deposit made by the sea before its final retreat. Sir Humphry Davy regards the soil or friable argillaceous surface of Great Britain at least, as having resulted from the disintegration of the rocky strata underneath.† There are doubtless but few rocks that would not be mouldered by exposure to the action of the atmosphere, and where the waters, before their final retreat, made no protecting layer, the argillaceous surface may be fairly referred to decomposition. But there is much reason for believing that the greater part of the *geest* of this country has subsided from fresh water, and is a true alluvion. It varies considerably, I acknowledge, in different places, but its essential constituents, clay, sand, and yellow oxide of iron, combined in varying proportions, seem still to be present. It is indeed destitute of the remains of aquatic animals, and might not therefore be supposed to have had the origin here intimated; but the old alluvial beds which have manifestly been accumulated by water, either salt or fresh, are almost equally destitute of aquatic exuviæ. On the other hand, it exhibits, in some places, an obscure stratification; it does not abound particularly in the undecomposed fragments of the rocks over which it is spread; its colour and density are nearly the same from bottom to top, and it envelopes, as we shall presently see, along with more or

* L'Histoire de la Terre et de l'Homme, tome v.

† See his Elements of Agricultural Chemistry.

less gravel, large masses of adventitious rocks, conditions that sufficiently characterize it as a distinct and real *formation.* It has been washed by the rains into the lower parts of the valleys, where, becoming blended with the recent alluvion of the streams, it encloses the bones of the arctic elephant, great mastodon, and other extinct quadrupeds. On the south side of the Ohio, it is only from four to eight or twelve feet deep; but to the north of that river, its thickness becomes so great, that the flœtz limestone is but here and there seen projecting through it.

The deposition of the *geest* seems to have been the last operation which the waters of the north performed upon this region; and was of course subsequent to the excavation of the valleys, as no deposit could have remained upon their acclivities, while the agent which formed them continued its action. You are, sir, already apprized, that to this formation belong the great blocks of foreign primitive, transition, and old flœtz rocks, which have excited in travellers so much astonishment, and which, in one point of resemblance at least, approximate the region south of Erie, Huron, Michigan, and the other lakes, so closely to that which stretches from the southern shores of the Baltic sea.

These masses, in the neighborhood of this place, are for the most part solitary; but in the interior of the State, it is not uncommon to find them grouped into heaps which are slightly covered with soil; and it is, I suspect, an aggregation of this kind, on one of the islands of Lake Huron, that a British officer mistook for granite *in place.** The size of these masses extends from that of gravel and pebbles to the diameter of eight or ten feet. The larger blocks are frequently found *upon* the old alluvial plains, but never, that I have understood, *within* them. Their geographical range is over the same region with the smaller foreign *debris* of our valleys, but more limited to the south-west. I have never seen a single block on the opposite side of the Ohio, and am not informed that any have been observed lower than the thirty-ninth degree of latitude.

I do not entertain a doubt that these fragments were enveloped in large fields of ice in a region far beyond the lakes, and floated hither by the same inundations that brought down and spread over the surface of this country, the *geest* in which they are imbedded. In the southern parts of this formation, they are not found; but this should be attributed to the influence of climate. The ice to which they were attached could not of course pass a certain latitude; and from the great increase of these masses as we advance towards the north, it would seem that many of the icebergs suffered dissolution long before they arrived at this *maximum.* Future observers will no doubt trace them to their parent strata in the arctic regions, as Von Buck has traced those which are lodged on the shores of the Baltic. The ice islands of the Atlantic ocean may reasonably be supposed to bring down, and deposit on its bed in the Temperate Zone, primordial masses, similar to those spread over some parts of this and the Europe-

* See Thompson's Annals of Phil. for March, 1816.

an continent. These islands are, I believe, not often seen further south than the forty-first degree, near two degrees north of their southern boundary here. This is probably attributable to the gulf stream; but for which the larger tracts of ice would undoubtedly attain as low a latitude as the southern limits of the primitive blocks in this country: and hence a probable conclusion may be drawn, that the temperature of the northern hemisphere has undergone but little change since the remote epoch when this part of the continent was for the last time subjected to inundation.

